# New-onset autoimmune disease after COVID-19

**DOI:** 10.3389/fimmu.2024.1337406

**Published:** 2024-02-08

**Authors:** Corrilynn O. Hileman, Shahdi K. Malakooti, Nirav Patil, Nora G. Singer, Grace A. McComsey

**Affiliations:** ^1^ Case Western Reserve University School of Medicine, Cleveland, OH, United States; ^2^ Department of Medicine, MetroHealth Medical Center, Cleveland, OH, United States; ^3^ University Hospitals Cleveland Medical Center, Cleveland, OH, United States

**Keywords:** autoimmune diseases, COVID-19, autoantibodies, risk factors, antinuclear antibodies

## Abstract

**Introduction:**

Severe acute respiratory syndrome coronavirus-2 (SARS-CoV-2) may trigger autoimmune disease (AD) through initial innate immune activation with subsequent aberrations in adaptive immune cells leading to AD. While there are multiple reports of incident AD diagnosed after COVID-19, the risk in the context of key circulating strains is unknown.

**Methods:**

TriNetX, a global, federated, health research network providing access to electronic medical records across 74 healthcare organizations, was utilized to define an adult cohort between January 1, 2020, and March 3, 2023. Exposure was defined as COVID-19 diagnosis (ICD-10 code or positive laboratory test). Age- and sex-propensity score-matched controls never had COVID-19 diagnosed. Outcomes were assessed 1 month to 1 year after the index date. Patients with AD prior to or within 1 month after the index date were excluded from the primary analysis. Incidence and risk ratios of each AD were assessed.

**Results:**

A total of 3,908,592 patients were included. Of 24 AD patients assessed, adjusted risk ratios for eight AD patients who had COVID-19 were higher compared to those who had no COVID-19. Cutaneous vasculitis (adjusted hazard ratio (aHR): 1.82; 95% CI 1.55–2.13), polyarteritis nodosa (aHR: 1.76; 95% CI 1.15–2.70), and hypersensitivity angiitis (aHR: 1.64; 95% CI 1.12–2.38) had the highest risk ratios. Overall, psoriasis (0.15%), rheumatoid arthritis (0.14%), and type 1 diabetes (0.13%) had the highest incidence during the study period, and of these, psoriasis and diabetes were more likely after COVID-19. The risk of any AD was lower if COVID-19 was diagnosed when Omicron variants were the predominant circulating strains. A positive antinuclear antibody was more likely and predictive of AD after COVID-19.

**Discussion:**

SARS-CoV-2 may be a potential trigger for some AD, but the risk for AD may decrease with time given the apparent lower risk after infection with Omicron variants.

## Introduction

Viral infections are often cited as important environmental triggers for autoimmune disease. In the setting of the global COVID-19 pandemic, this is highly relevant, as millions of individuals have been infected with severe acute respiratory syndrome coronavirus-2 (SARS-CoV-2). Indeed, there have been multiple reports of newly diagnosed autoimmune diseases after COVID-19 (1, 2). With the breadth of autoimmune disease manifestations, the rarity of many autoimmune diseases, and the lack of accumulated data in the context of COVID-19 variants up to this point, the overall risk of autoimmune disease after COVID-19 including recent key COVID-19 variants is not yet known.

The pathophysiology of autoimmune disease is complex, and the interplay of multiple factors, including genetic and environmental, likely contribute. Simplistically, the host immune response to viral infection has been postulated as a trigger for autoimmunity and includes the production of both interferons (especially alpha interferon), presentation of nuclear contents by “netting” neutrophils, and subsequent maturation of plasmacytoid dendritic cells that act as potent antigen-presenting cells. These virus-induced T cell-mediated autoimmune responses in the right host may lead to autoimmune disease *via* activation of the adaptive immune system resulting in B- and T-cell activations as evidenced first by autoantibodies and later by dysregulated T cells that contribute to overall loss of tolerance to self-antigen.

Interestingly, autoantibodies have been detected in patients with COVID-19 (3, 4). Further, some human proteins have homologous regions with SARS-CoV-2 peptides that could function as autoantigens (5). Additionally, it is clear that in some people with COVID-19, SARS-CoV-2 infection mediates a hyperinflammatory state. Dysregulated inflammasome activation has been implicated in autoimmune disease pathogenesis, and SARS-CoV-2 can activate the inflammasome (nod-like family, pyrin domain-containing 3, or NLRP3), which regulates the secretion of proinflammatory cytokines interleukin 1 beta (IL-1β) and IL-18 (6). More research is needed in this area; however, there is biological plausibility linking SARS-CoV-2 with autoimmunity.

The purpose of this study was to assess the risk of new-onset autoimmune disease within the first year after COVID-19 diagnosis in the context of the predominate circulating variants at the time of infection. We hypothesized that autoimmune disease diagnoses would be higher after COVID-19 infection than in age- and sex-matched controls and that risk would be attenuated when COVID-19 diagnosis occurred when the predominate circulating strains were the Omicron variants. While positive antinuclear antibodies (ANAs) are associated with a variety of autoimmune diseases, a positive ANA test alone is neither sufficient for rheumatologic diagnosis nor predictive of disease development. Therefore, our secondary aim was to evaluate the risk of ANA positivity after COVID-19 and how well ANA positivity predicted the development of new autoimmune diseases within the first year after COVID-19 diagnosis.

## Materials and methods

This was a retrospective and population-based cohort study utilizing TriNetX. TriNetX is a global, federated, health research network providing access to electronic medical records including diagnoses, procedures, medications, laboratory values, and genomic information across large healthcare organizations. TriNetX provides de-identified data, transformed into a proprietary data schema, including an extensive data quality and accuracy assessment. This analysis was performed on data drawn from 74 healthcare organizations and completed on March 3, 2023. The study population was defined as adults 18 years of age or older, seen on or after January 1, 2020, with at least one follow-up visit after the index date. Patients with any of the autoimmune diseases evaluated as outcomes in this study diagnosed prior to the index date or within 1 month after the index date were excluded from the primary analysis. The exposure of interest was COVID-19 diagnosis defined by ICD-10 code or positive laboratory test (see **Supplementary Table 1** for ICD-10 codes and laboratory tests included). Controls did not have COVID-19 diagnosis (defined by the same criteria) and were propensity score-matched to patients with COVID-19 by age and sex. The index date was defined as the date of COVID-19 diagnosis for the exposed group or first provider visit for any reason during the study period for controls. ANA positivity was defined as nuclear antibody presence in serum by immunofluorescence. This study was approved by the Institution Board Review Committee at Case Western Reserve University/University Hospitals Cleveland Medical Center (STUDY20231104). Written informed consent was waived, as the TriNetX system safeguards patients’ privacy in reporting de-identified data.

Outcomes, i.e., incident autoimmune diseases, selected for inclusion were those previously reported in case reports and case series as well as additional autoimmune diseases to attempt to develop as complete a list as possible. Outcomes were defined by ICD-10 codes (see **Supplementary Table 1** for ICD-10 codes utilized for each autoimmune disease included). Outcomes were assessed starting 1 month after the index date until 1 year after.

### Statistical analysis

The two groups, the exposed or COVID-19 group and the controls or no COVID-19 group, were propensity score-matched by age and sex. Demographics were described by frequency and percent for categorical variables and by mean ± standard deviation for continuous variables for each group. Incidence of each autoimmune disease and risk ratios were assessed for each outcome, i.e., patients with outcome/total patients per group with 95% confidence intervals. Incidence and risk ratios were adjusted for age and sex through propensity score matching as described above. In the primary analyses, patients with any of the autoimmune diseases evaluated as outcomes in this study diagnosed prior to the index date or within 1 month after the index date were excluded. As part of the secondary analyses, patients with the specific outcome being analyzed were excluded from the analysis for that outcome only. For the secondary analyses, the cohort was propensity score-matched by age and sex prior to excluding the patients with known disease.

## Results

Data were available from 1,954,296 adults from January 1, 2020, to March 3, 2023, who lacked prior autoimmune disease and who were diagnosed with COVID-19. Adults without prior autoimmune disease and a diagnosis of COVID-19 during the same time period were propensity score-matched by age and sex at birth to these adults to generate a cohort of 3,908,592 people. Overall, the mean age ± standard deviation (SD) was 48.7 ± 17.9, and 57.7% were women. There were more people from racial and ethnic minorities among those who had COVID-19; however, there were also more people with unknown race and/or ethnicity among those who did not have COVID-19 (see **Table 1**).

**Table 1:** Demographics overall and by COVID-19 exposure group.

	Overall N = 3,908,592	COVID-19 n = 1,954,296	No COVID-19 n = 1,954,296	p-Value
Age (years) at index	48.7 ± 17.9	48.7 ± 17.9	48.7 ± 17.9	>0.99
Sex, n (%)				
Female	2,253,498 (57.7%)	1,126,749 (57.7%)	1,126,749 (57.7%)	>0.99
Male	1,654,160 (42.3%)	827,080 (42.3%)	827,080 (42.3%)	>0.99
Unknown	934 (<1%)	467 (<1%)	467 (<1%)	>0.99
Race, n (%)				
White	2,171,935 (55.6%)	1,139,355 (58.3%)	1,032,581 (52.8%)	<0.001
Black/African American	516,815 (13.2%)	280,842 (14.4%)	235,973 (12.1%)	<0.001
Asian	94,298 (2.4%)	42,225 (2.2%)	52,073 (2.7%)	<0.001
American Indian, Alaskan Native	13,447 (0.3%)	7,038 (0.4%)	6,409 (0.3%)	<0.001
Pacific Islander	4,947 (0.1%)	2,654 (0.1%)	2,293 (0.1%)	<0.001
Unknown	1,107,149 (28.3%)	482,182 (24.7%)	624,967 (32%)	<0.001
Ethnicity, n (%)				
Not Hispanic/Latino	2,133,897 (54.6%)	1,143,516 (58.5%)	990,381 (50.7%)	<0.001
Hispanic/Latino	296,358 (7.6%)	171,282 (8.8%)	125,076 (6.4%)	<0.001
Unknown	1,478,337 (37.8%)	639,498 (32.7%)	838,839 (42.9%)	<0.001

Groups are matched by propensity score. Propensity scoring included age, male sex, and female sex. People with any prevalent autoimmune diseases prior to or within 1 month after the index date were excluded prior to propensity score matching. Values shown are mean ± standard deviation for continuous variables and frequency (column percent) for categorical variables.

### Risk of incident autoimmune disease after COVID-19

**Figure 1:**
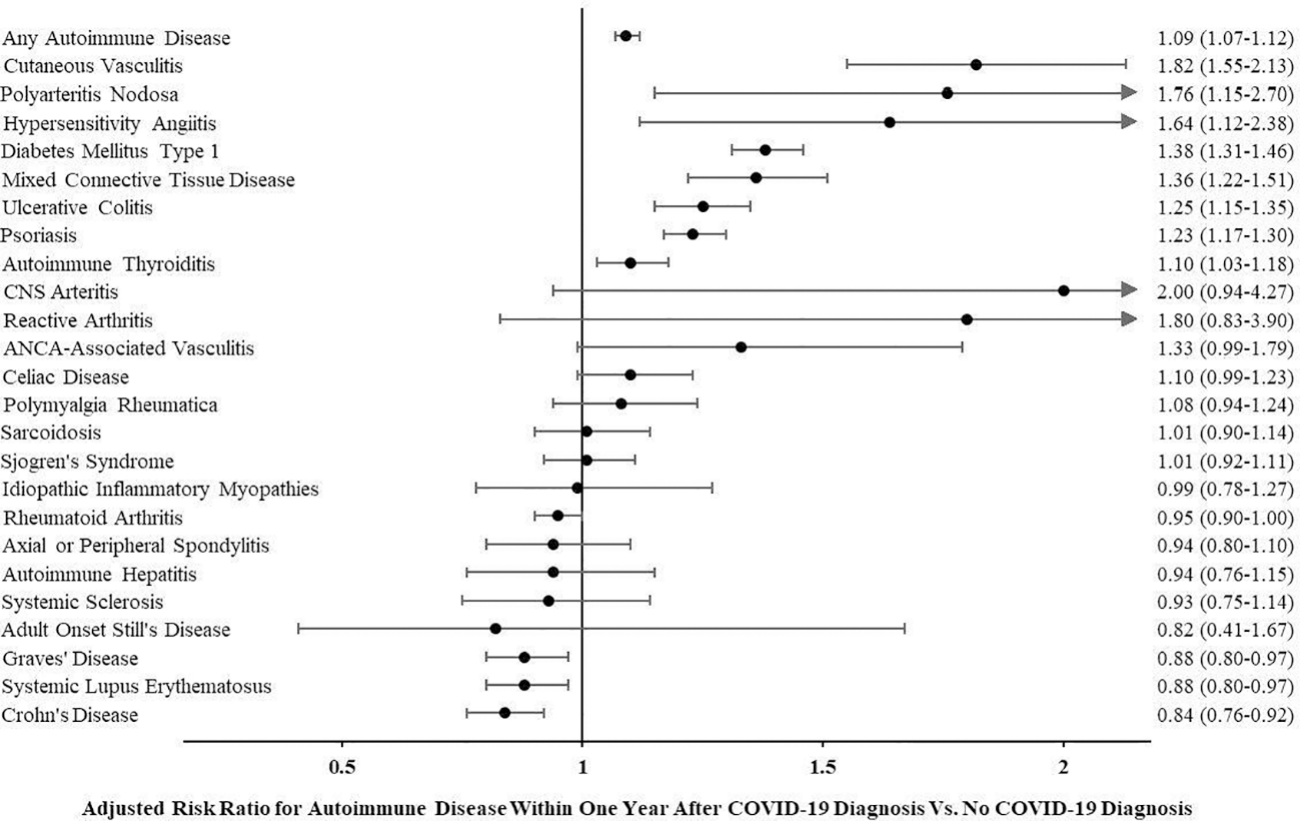
Adjusted risk ratio for autoimmune disease within 1 year after COVID-19 diagnosis *vs.* no COVID-19 diagnosis.

The risk of being diagnosed with any autoimmune disease was higher within 1 year following COVID-19 compared to a similar time period in age- and sex-matched controls who did not have COVID-19 diagnosis (adjusted risk ratio (aRR) for any autoimmune disease 1.09 (95% confidence interval or CI 1.07–1.12)). In evaluating each type of autoimmune disease individually, one-third (8 out of 24) of the autoimmune diseases assessed were more likely to be diagnosed after COVID-19. **Figure 1** shows adjusted risk ratios for each autoimmune disease assessed. Cutaneous vasculitis (aRR 1.82 (95% CI 1.55–2.13)), polyarteritis nodosa (aRR 1.76 (1.15–2.70)), and hypersensitivity angiitis (aRR 1.64 (1.12–2.38)) were associated with the highest risk. The three autoimmune diseases with the highest incidence during the study period were psoriasis (diagnosed in 5,690 or 0.15%), rheumatoid arthritis (5,618 or 0.14%), and type 1 diabetes mellitus (5,015 or 0.13%). Of these, both psoriasis (aRR 1.23 (95% CI 1.17–1.30)) and type 1 diabetes mellitus (aRR 1.38 (1.31–1.46)) were more common after COVID-19. Graves’ disease (0.88 (0.80–0.97)), systemic lupus erythematosus (0.88 (0.80–0.97)), and Crohn’s disease (0.84 (0.76–0.92)) were the only diseases less likely to be diagnosed after COVID-19. See **Table 2** for the incidence of each autoimmune disease assessed overall as well as by group and adjusted risk ratios.

**Table 2:** Incident autoimmune diseases overall and by COVID-19 exposure group.

	Overall N = 3,908,592	COVID-19 n = 1,954,296	No COVID-19 n = 1,954,296	Adjusted risk ratio (95% CI)
Any autoimmune disease	31,052 (0.794%)	16,199 (0.829%)	14,853 (0.760%)	1.09 (1.07–1.12)
Autoimmune diseases more likely after COVID-19				
Cutaneous vasculitis	674 (0.017%)	435 (0.022%)	239 (0.012%)	1.82 (1.55–2.13)
Polyarteritis nodosa	91 (0.002%)	58 (0.003%)	33 (0.002%)	1.76 (1.15–2.70)
Hypersensitivity angiitis	116 (0.003%)	72 (0.004%)	44 (0.002%)	1.64 (1.12–2.38)
Type 1 diabetes mellitus	5,014 (0.128%)	2,908 (0.149%)	2,106 (0.108%)	1.38 (1.31–1.46)
Mixed connective tissue disease	1,407 (0.036%)	811 (0.041%)	596 (0.030%)	1.36 (1.22–1.51)
Ulcerative colitis	2,447 (0.063%)	1,359 (0.070%)	1,088 (0.056%)	1.25 (1.15–1.35)
Psoriasis	5,690 (0.146%)	3,137 (0.161%)	2,553 (0.131%)	1.23 (1.17–1.30)
Autoimmune thyroiditis	3,625 (0.093%)	1,902 (0.097%)	1,723 (0.088%)	1.10 (1.03–1.18)
Autoimmune diseases less likely after COVID-19				
Graves’ disease	1,524 (0.039%)	713 (0.036%)	811 (0.041%)	0.88 (0.80–0.97)
Systemic lupus erythematosus	1,596 (0.041%)	746 (0.038%)	850 (0.043%)	0.88 (0.80–0.97)
Crohn’s disease	1,737 (0.044%)	792 (0.041%)	945 (0.048%)	0.84 (0.76–0.92)
Autoimmune diseases with no associated increased or decreased risk after COVID-19				
CNS arteritis	30 (0.001%)	20 (0.001%)	≤10 (0.001%)	2.00 (0.94–4.27)
Reactive arthritis	28 (0.001%)	18 (0.001%)	≤10 (0.001%)	1.80 (0.83–3.90)
ANCA associated vasculitis	177 (0.005%)	101 (0.005%)	76 (0.004%)	1.33 (0.99–1.79)
Celiac disease	1,313 (0.034%)	689 (0.035%)	624 (0.032%)	1.10 (0.99–1.23)
Polymyalgia rheumatica	834 (0.021%)	433 (0.022%)	401 (0.021%)	1.08 (0.94–1.24)
Sarcoidosis	1,129 (0.029%)	568 (0.029%)	561 (0.029%)	1.01 (0.90–1.14)
Sjögren’s syndrome	1,811 (0.046%)	910 (0.047%)	901 (0.046%)	1.01 (0.92–1.11)
Idiopathic inflammatory myopathies	261 (0.007%)	130 (0.007%)	131 (0.007%)	0.99 (0.78–1.27)
Rheumatoid arthritis	5,618 (0.144%)	2,740 (0.140%)	2,878 (0.147%)	0.95 (0.90–1.00)
Axial or peripheral spondylitis	616 (0.016%)	298 (0.015%)	318 (0.016%)	0.94 (0.80–1.10)
Autoimmune hepatitis	370 (0.009%)	179 (0.009%)	191 (0.010%)	0.94 (0.76–1.15)
Systemic sclerosis	358 (0.009%)	172 (0.009%)	186 (0.010%)	0.93 (0.75–1.14)
Adult-onset Still’s disease	31 (0.001%)	14 (0.001%)	17 (0.001%)	0.82 (0.41–1.67)

Groups are matched by propensity score. Propensity scoring included age, male sex, and female sex. People with any prevalent autoimmune diseases prior to or within 1 month after the index date were excluded from this analysis prior to propensity score matching.

CNS, central nervous system; ANCA, anti-neutrophil cytoplasmic antibodies.

Of those with COVID-19, the risk of having been hospitalized within 10 days of COVID-19 diagnosis was higher for people who developed autoimmune disease after COVID-19 than people who did not (aRR for hospitalization 1.54 (95% CI 1.44–1.63)) (see **Supplementary Figure 1**).

In the secondary analysis, people with a specific autoimmune disease prior to or within 1 month after the index date were excluded from the analysis for that outcome. Overall, 4,407,892 individuals were included in this cohort. **Supplementary Table 2** shows demographics overall and by COVID-19 exposure group, which were similar to the primary analysis. In this analysis, the risk of being diagnosed with 18 out of the 24 autoimmune diseases evaluated was higher during the 1 year after COVID-19 diagnosis than during a similar time period in controls. The other autoimmune diseases had similar incidences over 1 year in both groups. See **Supplementary Table 3** for the incidence of each autoimmune disease assessed overall and by group with adjusted risk ratios.

### Effect of different timeframes on incident autoimmune disease risk after COVID-19

**Figure 2:**
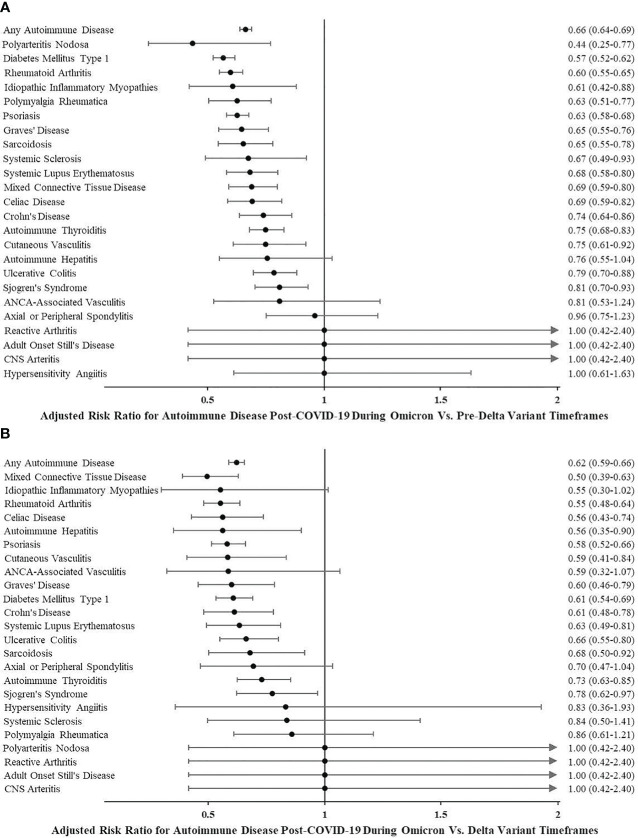
**(A)** Adjusted risk ratio for autoimmune disease post-COVID-19 during Omicron *vs.* pre-Delta variant timeframes. **(B)** Adjusted risk ratio for autoimmune disease post-COVID-19 during Omicron *vs.* Delta variant timeframes.

People diagnosed with COVID-19 from July 1, 2021, to November 30, 2021 (during which time the predominant circulating strain of SARS-CoV-2 was the Delta variant), as well as people diagnosed from January 1, 2020, to June 30, 2021 (pre-Delta variant timeframe), had a higher risk of any autoimmune disease when compared to people diagnosed with COVID-19 on or after December 1, 2021. Following December 1, 2021, Omicron SARS-CoV-2 variants were the predominant circulating strains in the USA. The adjusted risk ratio was 0.62 (95% CI 0.59–0.66) for incident autoimmune disease during Omicron *vs.* Delta variant timeframes and 0.66 (95% CI 0.64–0.69) during Omicron *vs.* pre-Delta variant timeframes. See **Figure 2** for autoimmune diseases more commonly diagnosed during Delta and pre-Delta than Omicron variant timeframes. None of the autoimmune diseases were more likely to be diagnosed in the first year following COVID-19 infection when the predominant circulating strains were the Omicron variants.

### The association of positive ANA test and incident autoimmune disease after COVID-19

In those without a history of autoimmune disease or a positive ANA test, the risk of having a positive ANA test was higher after COVID-19 (980 out of 1,949,921) than for those who did not have COVID-19 (578 out of 1,949,921), adjusting for age and sex (adjusted risk ratio 1.70 (95% CI 1.53–1.88)). Among those with COVID-19, the risk of developing an autoimmune disease was higher for those with a positive ANA test after COVID-19 diagnosis than those without a positive ANA test after adjusting for age and sex (adjusted risk ratio 11.90 (95% CI 6.28–22.55)) (see **Supplementary Figure 2** for flowchart with absolute numbers). Specifically, a positive ANA test after COVID-19 was predictive of a new diagnosis for each of the following autoimmune diseases: systemic lupus erythematosus, rheumatoid arthritis, mixed connective tissue disease, Sjögren’s syndrome, cutaneous vasculitis, hypersensitivity angiitis, autoimmune thyroiditis, Graves’ disease, Crohn’s disease, celiac disease, polymyalgia rheumatica, idiopathic inflammatory myopathies, autoimmune hepatitis, and systemic sclerosis (see **Table 3**).

**Table 3:** Risk of autoimmune disease by ANA status.

	Positive ANA n = 991	Negative or no ANA n = 991	Adjusted risk ratio (95% CI)
Any autoimmune disease	119 (12.000%)	≤10 (1.009%)	11.90 (6.28–22.55)
Systemic lupus erythematosus	28 (2.825%)	0 (0%)	–
Rheumatoid arthritis	32 (3.229%)	≤10 (1.009%)	3.20 (1.58–6.47)
Mixed connective tissue disease	19 (1.917%)	0 (0%)	–
Sjögren’s syndrome	17 (1.715%)	0 (0%)	–
Cutaneous vasculitis	≤10 (1.009%)	0 (0%)	–
Hypersensitivity angiitis	≤10 (1.009%)	0 (0%)	–
Autoimmune thyroiditis	≤10 (1.009%)	0 (0%)	–
Graves’ disease	≤10 (1.009%)	0 (0%)	–
Crohn’s disease	≤10 (1.009%)	0 (0%)	–
Celiac disease	≤10 (1.009%)	0 (0%)	–
Polymyalgia rheumatica	≤10 (1.009%)	0 (0%)	–
Idiopathic inflammatory myopathies	≤10 (1.009%)	0 (0%)	–
Autoimmune hepatitis	≤10 (1.009%)	0 (0%)	–
Systemic sclerosis	≤10 (1.009%)	0 (0%)	–
Type 1 diabetes mellitus	≤10 (1.009%)	≤10 (1.009%)	1 (0.42–2.39)
Ulcerative colitis	≤10 (1.009%)	≤10 (1.009%)	1 (0.42–2.39)
Psoriasis	≤10 (1.009%)	≤10 (1.009%)	1 (0.42–2.39)
Sarcoidosis	≤10 (1.009%)	≤10 (1.009%)	1 (0.42–2.39)
Axial or peripheral spondylitis	≤10 (1.009%)	≤10 (1.009%)	1 (0.42–2.39)
Polyarteritis nodosa	0 (0%)	0 (0%)	–
CNS arteritis	0 (0%)	0 (0%)	–
Reactive arthritis	0 (0%)	0 (0%)	–
ANCA associated vasculitis	0 (0%)	0 (0%)	–
Adult-onset Still’s disease	0 (0%)	0 (0%)	–

Groups are matched by propensity score. Propensity scoring included age, male sex, and female sex. People with any prevalent autoimmune diseases or positive ANA test prior to or within 1 month after the index date were excluded from this analysis prior to propensity score matching.

ANA, antinuclear antibody; CNS, central nervous system; ANCA, anti-neutrophil cytoplasmic antibodies.

### Effect of any COVID-19 vaccination on incident autoimmune disease risk after COVID-19

Of 1,953,971 patients with COVID-19 and without a history of autoimmune disease, 159,306 (8.2%) had documentation of any COVID-19 vaccination in the TriNetX database. The adjusted risk ratio of any new autoimmune disease diagnosis within 1 year of the index date was 1.18 (95% CI 1.10–1.27) for those who received vaccination *vs.* those with no documentation of vaccination (see **Supplementary Figure 3** for flowchart with absolute numbers). In assessing this for each separate autoimmune disease, increased risk post-COVID-19 in those vaccinated compared to those with no documentation of vaccination was only apparent for celiac disease (adjusted risk ratio 1.80 (95% CI 1.22–2.65)), autoimmune thyroiditis (1.70 (1.37–2.11)), Sjögren’s syndrome (1.54 (1.16–2.04)), psoriasis (1.42 (1.21–1.66)), and ulcerative colitis (1.40 (1.09–1.80)). The risk of polymyalgia rheumatica was similar regardless of vaccination status. No autoimmune disease was less common post-COVID-19 in those who received vaccination when compared with those with no documentation of vaccination. See **Supplementary Table 4** for the incidence of each autoimmune disease assessed overall and by group and adjusted risk ratios.

## Discussion

This is the first study of this magnitude of incident autoimmune disease including timeframes where circulating SARS-CoV-2 strains including more recent Omicron variants predominated. We demonstrate that COVID-19 diagnosis was associated with an increased risk of autoimmune disease in the year after infection, and notably, a positive ANA test was more likely after COVID-19 and predicted risk of new-onset autoimmune diseases.

Our finding of an increase in cutaneous vasculitis and polyarteritis after COVID-19 infection is not unexpected given that cutaneous small-vessel vasculitis or capillaritis such as leukocytoclastic vasculitis is frequently associated with perinuclear anti-neutrophil cytoplasmic antibodies (p-ANCA) and antibodies against myeloperoxidase (anti-MPO) and is seen after a variety of infections (streptococcal and hepatitis infection in Henoch–Schonlein purpura) and environmental insults (levamisole in therapeutic and illicit drugs) (7, 8). These diseases also may be accompanied by autoantibodies to cytoplasmic ANCA (c-ANCA) as well as anti-phospholipid antibodies (a major cause of clots following COVID-19 infection). Cutaneous and systemic polyarteritis have both been reported in association with genetic deficiency of adenosine deaminase-2 (DADA2). Adenosine deaminase-2 (ADA2) function(s) are not entirely known, but the protein does appear to contribute to vascular integrity. High levels of ADA2 have been reported in association with infectious and inflammatory illnesses (9) including macrophage activation syndrome in systemic-onset juvenile idiopathic arthritis (10). DADA2 also has been associated with the activation of alpha interferon-associated genes, but any interrelationship between these two states has not been described yet in COVID-19.

Of those with COVID-19, the risk of having been hospitalized within 10 days of COVID-19 diagnosis was higher for people who developed autoimmune disease after COVID-19 than people who did not develop autoimmune disease. This suggests that those who developed autoimmune disease may have had more severe manifestations of COVID-19 than people who did not develop autoimmune disease. Further, prior to vaccination and treatment availability, individuals with genetic risk factors for systemic lupus erythematosus (similar to those with pre-formed anti-cytokine antibodies) may have been at increased risk of life-threatening COVID-19 infection and mortality, potentially resulting in the underrepresentation of systemic lupus erythematosus in COVID-19 survivors when analyzing later timeframes in context of predominant SARS-CoV-2 circulating strains.

The effect of differing circulating strains on the advent of post-acute sequelae of SARS-CoV-2 (PASC) has been previously investigated. Whether PASC is defined by the persistence of symptoms months after a COVID-19 infection or by new-onset health conditions linked to COVID-19, such as new-onset diabetes and cardiovascular disease, Omicron variants appear to be associated with lesser risk than earlier strains (11–15). Our study extends these observations of the potentially less pathogenic nature of Omicron variants to new-onset autoimmune diseases following COVID-19.

Another interesting observation in our study is the apparent increased risk of certain autoimmune diseases after vaccination. In contrast to our observation of autoimmune disease, studies have shown that vaccination is protective against PASC symptoms and incident diabetes after COVID-19 infection (11, 16, 17). New-onset autoimmune phenomena have been described post-COVID-19 vaccination (including immune-mediated hepatitis after COVID-19 vaccination), not all of which have a clear causal relationship established (18–20). Using real-world electronic health record data is more prone to underreporting of vaccination status, which may explain the low vaccination numbers in our study. That said, more studies are needed to better define the risk of autoimmune disease after vaccination.

The finding that ANA positivity is more common after COVID-19 infection and is predictive of new-onset autoimmune disease is noteworthy. In contrast to the often transient positivity of antiphospholipid antibodies, p-ANCA, anti-MPO, and autoantibodies to rheumatoid arthritis and systemic lupus erythematosus may be present for 8 years or more prior to the onset of incident autoimmune disease (21, 22). This implies that if autoantibodies are present at increased frequency, the incidence of autoantibody disease may rise over longer periods of time, and our estimates of the frequency of autoimmunity may vastly underestimate the effect of COVID-19 on incident autoimmunity in long-term studies. Further, if Omicron variants overly induce lower levels of innate immune activation and subsequently less stimulation of B and T cells, it may take longer to induce similar levels of autoantibodies and T-cell derangements. It is therefore impossible to exclude the possibility that there will be a longer lag in the onset of new autoimmune disease following infection with the Omicron variants compared to Alpha/Delta SARS-CoV-2 and that ultimately, the rates of autoimmune disease may be similar to those seen with all the SARS-CoV-2 variants. Our report undoubtedly includes some patients in the control group who were asymptomatic for COVID-19 and who were neither tested for COVID-19 nor recognized as having COVID-19. This could lead to type II error, as some patients who developed COVID-19-related autoimmune disease may have been misclassified as having been COVID-19 uninfected, leading to smaller effect sizes regarding the risk of autoimmune disease after COVID-19. Importantly, our study also differs from prior reports from TriNetX that required either a positive or negative polymerase chain reaction test to be available for the analyses and focused only on the pre-Omicron era of COVID-19 (January 2020–December 2021) (23). In that way, our results are more generalizable, as they reflect the aggregation of the effects of pre-Delta, Delta, and Omicron variants of COVID-19 with comparisons for incident autoimmune disease and can be re-run at intervals for many years to come.

In addition, ANAs have been classified historically using indirect immunofluorescence assays (IFAs) mostly on the human epidermoid carcinoma (Hep2) cell line to detect nuclear localization, and ANA by IFA was used to define ANA positivity in our study. However, many laboratories have switched to a multiplex assay to measure autoantibodies directly by the target antigen. Clinicians may conclude that an ANA is positive when autoantibodies measure an antigen in the cocktail and may or may not obtain concomitant or subsequent ANA by IFA on the Hep2 cell line to detect nuclear autoantibodies. This is important, as multiplex assays may result in overdiagnosis of autoimmune disease based on a single autoantibody specificity, as positive autoantibody status is sometimes equated to a clinical diagnosis of autoimmunity by non-rheumatologists. This is particularly relevant to anti-U1-ribonucleoprotein (anti-RNP), which accompanies a high-titer ANA by IFA for classification as mixed connective tissue disease but is seen frequently at low levels in the current multiplex technology used across multiple centers. The specificity of low-titer reactivity by multiplex as predictive of future autoimmune disease has never been established, but such low-titer antibodies are observed frequently after COVID-19. Whether any of the anti-SARS-CoV-2 protein antibodies cross-react with antigens in the multiplex assays and therefore wane over time also is a topic ripe for exploration.

A strength of this analysis included the use of TriNetX to analyze data from a large population encompassing 74 healthcare organizations throughout the globe. However, we should recognize that we were unable to adjust for all potential confounders. Limitations of our study are similar to other large studies using electronic health record-derived data and include reliance on ICD-10 diagnoses for disease classification, which may have led to some misclassification. Similarly, we relied on electronic health record data for diagnosis of COVID-19 infection, which may have misclassified some asymptomatic COVID-19 infections as uninfected controls. However, if the latter is true, the effect of COVID-19 infection on incident autoimmune disease may have been underestimated. Further, as with all studies assessing new health conditions after COVID-19, we cannot rule out the possibility that some apparent incident autoimmune diseases were actually flares of previously undiagnosed disease, nor can we rule out potential relation with disproportionate stress. Finally, while Omicron may relate to reduced pathogenesis regardless of vaccination and prior infection status when compared to Delta (23), the role of innate and adaptive immunity in new-onset autoimmune disease after COVID-19 in the context of key variants is yet to be determined. Despite these limitations, however, the major strengths of our study lie in the fact that we have carefully captured the emergence of new-onset autoimmune disease following COVID-19 in a large-scale study. Importantly, our study differs from a prior report from TriNetX that required either a positive or negative polymerase chain reaction test to be included in the analyses and focused only on the pre-Omicron era of COVID-19 (January 2020–December 2021) (24), whereas our report reflects comparison and aggregation of the effects of pre-Delta, Delta, and Omicron variants of COVID-19 on incident autoimmune disease.

In summary, several autoimmune diseases were more likely to be diagnosed within the first year after COVID-19 than in age- and sex-matched controls. The risk of new-onset autoimmune diseases after COVID-19 appears to be attenuated with the more recent Omicron strains. Positive ANA test is more common after COVID-19 and is predictive of incident autoimmune diseases. This suggests that SARS-CoV-2 may be a trigger for certain autoimmune diseases. Future work must focus on longer-term observational cohorts and should assess the persistence and predictive value of different measured autoantibodies.

## Data availability statement

The original contributions presented in the study are included in the article/supplementary material. Further inquiries can be directed to the corresponding authors.

## Ethics statement

The studies involving humans were approved by Institution Board Review committee at Case Western Reserve University/University Hospitals Cleveland Medical Center (STUDY20231104). The studies were conducted in accordance with the local legislation and institutional requirements. The ethics committee/institutional review board waived the requirement of written informed consent for participation from the participants or the participants’ legal guardians/next of kin because data from the TriNetX system safeguards patient’s privacy in reporting deidentified data.

## Author contributions

CH: Writing – original draft, Writing – review & editing. SM: Writing – original draft, Writing – review & editing. NP: Data curation, Formal analysis, Writing – review & editing. NS: Supervision, Writing – review & editing. GM: Conceptualization, Supervision, Writing – review & editing.
