# Supplementary figures and images for: New-onset autoimmune disease after COVID-19
Source: Front Immunol. 2024 Feb 8;15:1337406. doi: 10.3389/fimmu.2024.1337406 (PMC10883027; doi:10.3389/fimmu.2024.1337406)

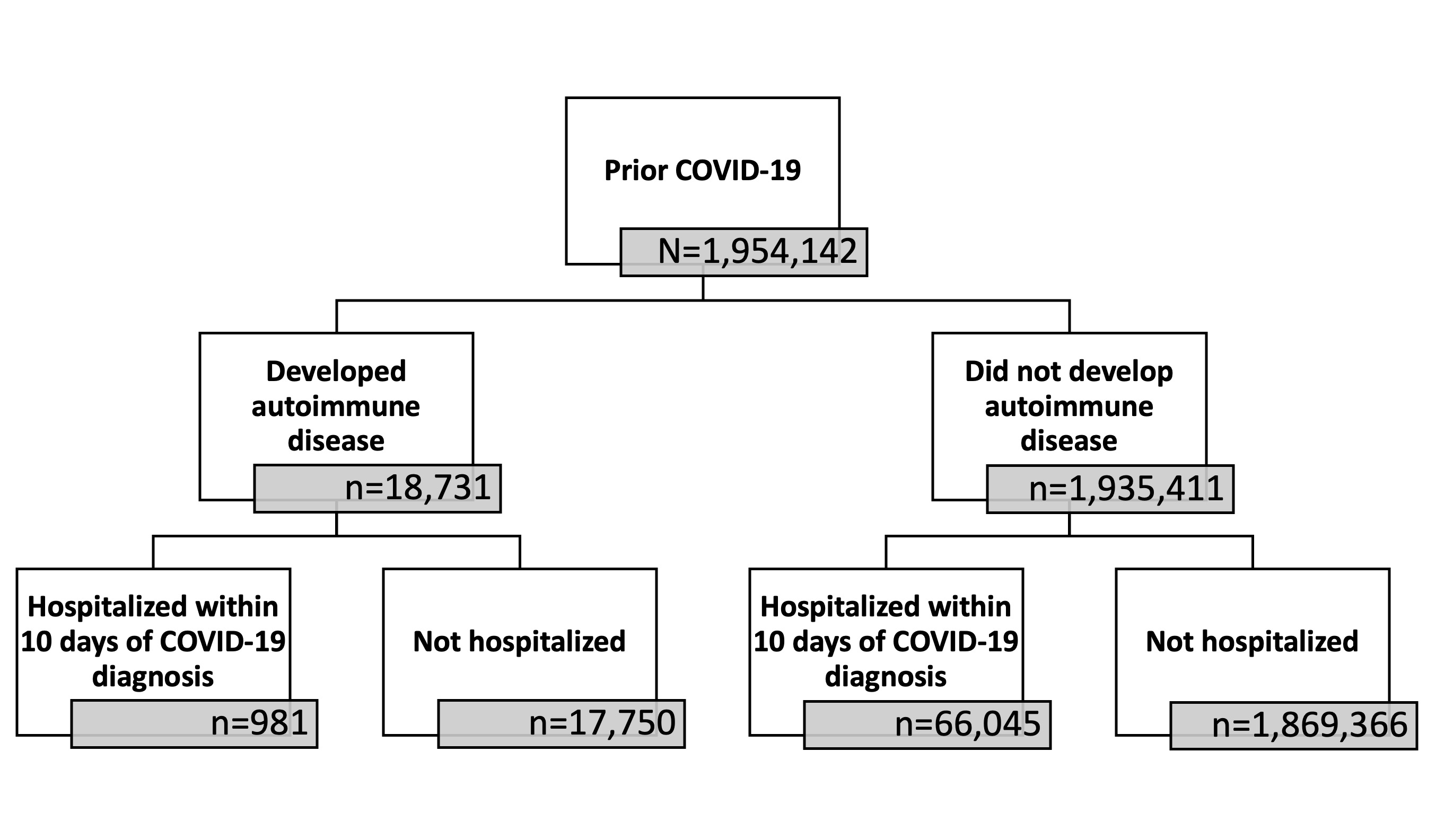

Supplement: Supplementary file 1 [file Image_1.jpeg]

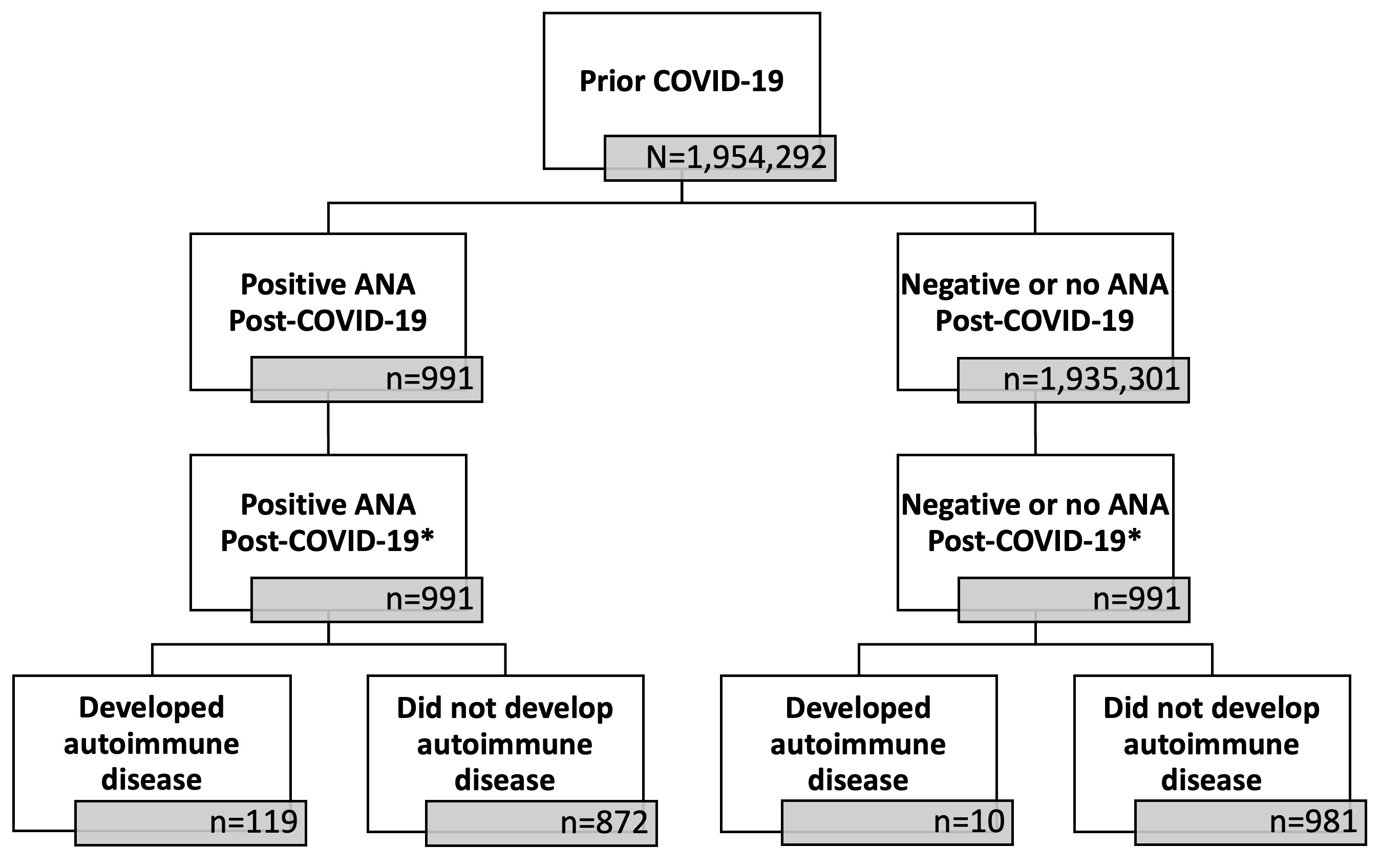

Supplement: Supplementary file 2 [file Image_2.jpeg]

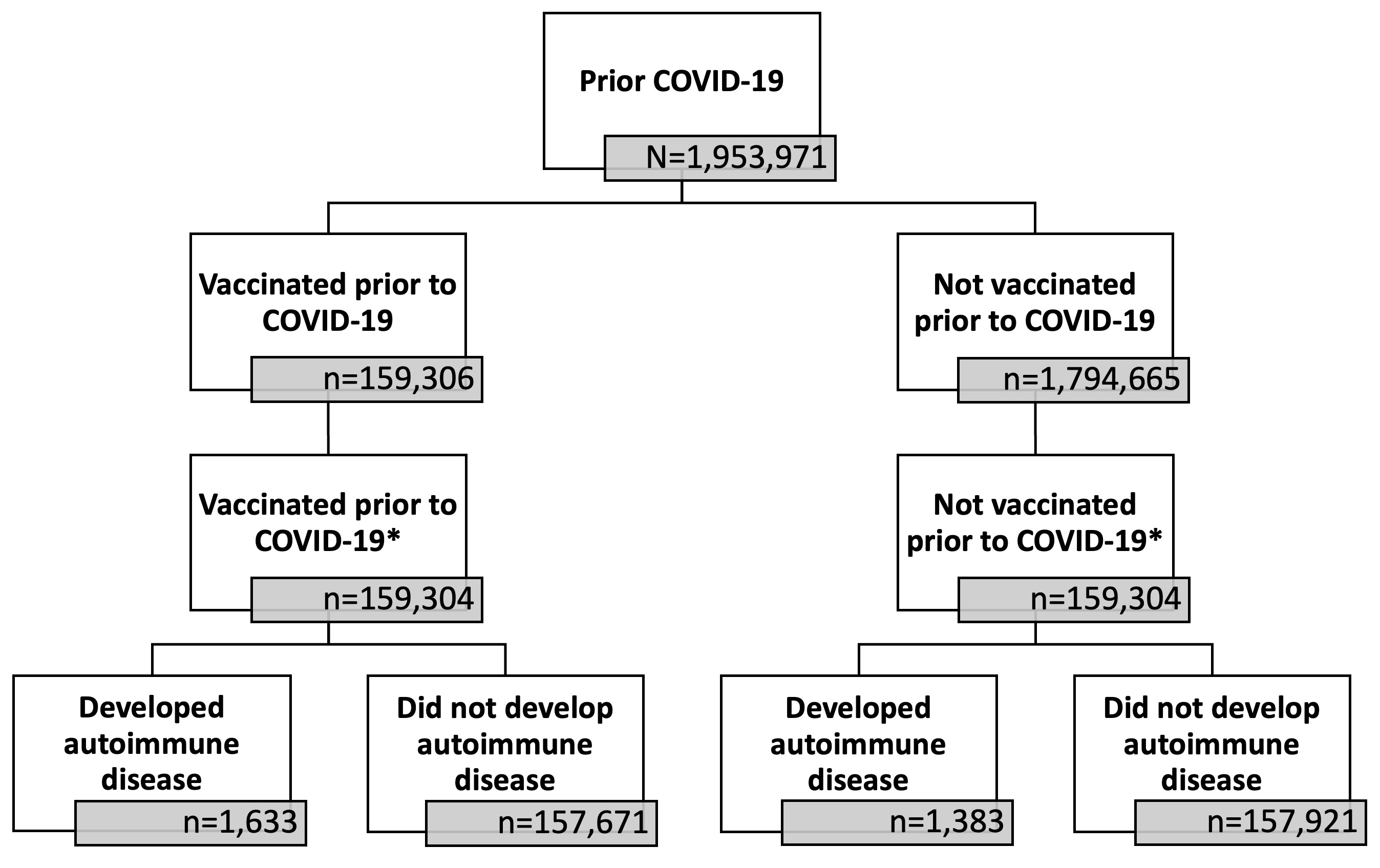

Supplement: Supplementary file 3 [file Image_3.jpeg]
