# Supplementary material for: New-onset autoimmune disease after COVID-19
Source: Front Immunol. 2024 Feb 8;15:1337406. doi: 10.3389/fimmu.2024.1337406 (PMC10883027; doi:10.3389/fimmu.2024.1337406)
Supplement: Supplementary file 4 [file Table_1.docx]

### **Supplemental Table 1. ICD-10 and Laboratory Codes.**

### **COVID Group Criteria.**

| Group 1 | | | | | |
| --- | --- | --- | --- | --- | --- |
|  | **Group 1A COVID** | | | | |
|  | must have | any of | diagnosis | ICD10CM:U07.1 | COVID-19 (at least 18 years old at event) |
|  |  |  | diagnosis | ICD10CM:J12.82 | Pneumonia due to COVID-19 (at least 18 years old at event) |
|  |  |  | diagnosis | ICD10CM:U07.2 | COVID-19, virus not identified (WHO) (at least 18 years old at event) |
|  |  |  | laboratory | TNX:9088 | SARS coronavirus 2 and related RNA [Presence] (labResult: Positive; at least 18 years old at event) |
|  |  |  | laboratory | LNC:94558-4 | SARS coronavirus 2 Ag [Presence] in Respiratory specimen by Rapid immunoassay (labResult: Positive; at least 18 years old at event) |
|  |  |  | laboratory | LNC:95209-3 | SARS coronavirus+SARS coronavirus 2 Ag [Presence] in Respiratory specimen by Rapid immunoassay (labResult: Positive; at least 18 years old at event) |
|  |  |  | laboratory | LNC:96119-3 | SARS-CoV-2 (COVID-19) Ag [Presence] in Upper respiratory specimen by Immunoassay (at least 18 years old at event; labResult: Positive) |
|  |  |  | laboratory | LNC:94763-0 | SARS-CoV-2 (COVID-19) [Presence] in Unspecified specimen by Organism specific culture (labResult: Positive; at least 18 years old at event) |
|  |  |  | laboratory | LNC:97097-0 | SARS-CoV-2 (COVID-19) Ag [Presence] in Upper respiratory specimen by Rapid immunoassay (labResult: Positive; at least 18 years old at event) |
|  | date constraint | | The terms in this group occurred on or after Jan 1, 2020 | | |
|  | event relationship | | Any instance of Group 1B occurred at least 1 day after the first instance of COVID | | |
|  | **Group 1B** | | | | |
|  | must have |  | visit | TNX:Visit | Visit (at least 18 years old at event) |

### **No COVID group criteria**

| Group 1 | | | | | |
| --- | --- | --- | --- | --- | --- |
|  | **Group 1A VISIT** | | | | |
|  | must have |  | visit | TNX:Visit | Visit (at least 18 years old at event) |
|  | date constraint | | The terms in this group occurred on or after Jan 1, 2020 | | |
|  | event relationship | | Any instance of No COVID occurred on or after the first instance of VISIT | | |
|  | **Group 1B No COVID** | | | | |
|  | cannot have |  | diagnosis | ICD10CM:U07.1 | COVID-19 (at least 18 years old at event) |
|  |  | or | diagnosis | ICD10CM:J12.82 | Pneumonia due to COVID-19 (at least 18 years old at event) |
|  |  | or | laboratory | TNX:9088 | SARS coronavirus 2 and related RNA [Presence] (labResult: Positive; at least 18 years old at event) |
|  |  | or | diagnosis | ICD10CM:U07.2 | COVID-19, virus not identified (WHO) (at least 18 years old at event) |
|  |  | or | laboratory | LNC:94558-4 | SARS coronavirus 2 Ag [Presence] in Respiratory specimen by Rapid immunoassay (labResult: Positive; at least 18 years old at event) |
|  |  | or | laboratory | LNC:95209-3 | SARS coronavirus+SARS coronavirus 2 Ag [Presence] in Respiratory specimen by Rapid immunoassay (labResult: Positive; at least 18 years old at event) |
|  |  | or | laboratory | LNC:94763-0 | SARS-CoV-2 (COVID-19) [Presence] in Unspecified specimen by Organism specific culture (labResult: Positive; at least 18 years old at event) |
|  |  | or | laboratory | LNC:96119-3 | SARS-CoV-2 (COVID-19) Ag [Presence] in Upper respiratory specimen by Immunoassay (at least 18 years old at event; labResult: Positive) |
|  |  | or | laboratory | LNC:97097-0 | SARS-CoV-2 (COVID-19) Ag [Presence] in Upper respiratory specimen by Rapid immunoassay (labResult: Positive; at least 18 years old at event) |
| Group 2 | | | | | |
|  | **Group 2A VISIT** | | | | |
|  | must have |  | visit | TNX:Visit | Visit (at least 18 years old at event) |
|  | date constraint | | The terms in this group occurred on or after Jan 1, 2020 | | |
|  | event relationship | | Any instance of VISIT2 occurred at least 1 day after the first instance of VISIT | | |
|  | **Group 2B VISIT2** | | | | |
|  | must have |  | visit | TNX:Visit | Visit (at least 18 years old at event) |

### **Autoimmune Conditions Definitions**

| Rheumatoid Arthritis | | | | |
| --- | --- | --- | --- | --- |
|  | **Outcome definition** | | | |
|  | | Diagnosis | ICD10CM:M05 | Rheumatoid arthritis with rheumatoid factor |
|  | | Diagnosis | ICD10CM:M06 | Other rheumatoid arthritis |
|  | **Settings for the performed analyses** | | | |
|  | | Risk analysis | | excluding patients with outcome prior to the time window |
| Axial or Peripheral Spondylitis | | | | |
|  | **Outcome definition** | | | |
|  | | Diagnosis | ICD10CM:M45 | Ankylosing spondylitis |
|  | **Settings for the performed analyses** | | | |
|  | | Risk analysis | | excluding patients with outcome prior to the time window |
| Reactive Arthritis | | | | |
|  | **Outcome definition** | | | |
|  | | Diagnosis | ICD10CM:M02.8 | Other reactive arthropathies |
|  | | Diagnosis | ICD10CM:M02.9 | Reactive arthropathy, unspecified |
|  | **Settings for the performed analyses** | | | |
|  | | Risk analysis | | excluding patients with outcome prior to the time window |
| Adult Onset Still Disease | | | | |
|  | **Outcome definition** | | | |
|  | | Diagnosis | ICD10CM:M06.1 | Adult-onset Still's disease |
|  | **Settings for the performed analyses** | | | |
|  | | Risk analysis | | excluding patients with outcome prior to the time window |
| Polymyalgia Rheumatica | | | | |
|  | **Outcome definition** | | | |
|  | | Diagnosis | ICD10CM:M35.3 | Polymyalgia rheumatica |
|  | | Diagnosis | ICD10CM:M35.5 | Multifocal fibrosclerosis |
|  | | Diagnosis | ICD10CM:M35.6 | Relapsing panniculitis [Weber-Christian] |
|  | **Settings for the performed analyses** | | | |
|  | | Risk analysis | | excluding patients with outcome prior to the time window |
| Polyarteritis Nodosa | | | | |
|  | **Outcome definition** | | | |
|  | | Diagnosis | ICD10CM:M30 | Polyarteritis nodosa and related conditions |
|  | **Settings for the performed analyses** | | | |
|  | | Risk analysis | | excluding patients with outcome prior to the time window |
| CNS Arteritis | | | | |
|  | **Outcome definition** | | | |
|  | | Diagnosis | ICD10CM:I67.7 | Cerebral arteritis, not elsewhere classified |
|  | **Settings for the performed analyses** | | | |
|  | | Risk analysis | | excluding patients with outcome prior to the time window |
| ANCA Associated Vasculitis | | | | |
|  | **Outcome definition** | | | |
|  | | Diagnosis | ICD10CM:M31.7 | Microscopic polyangiitis |
|  | | Diagnosis | ICD10CM:M31.3 | Wegener's granulomatosis |
|  | **Settings for the performed analyses** | | | |
|  | | Risk analysis | | excluding patients with outcome prior to the time window |
| Cutaneous Vasculitis | | | | |
|  | **Outcome definition** | | | |
|  | | Diagnosis | ICD10CM:L95 | Vasculitis limited to skin, not elsewhere classified |
|  | **Settings for the performed analyses** | | | |
|  | | Risk analysis | | excluding patients with outcome prior to the time window |
| Systemic Lupus Erythematosus | | | | |
|  | **Outcome definition** | | | |
|  | | Diagnosis | ICD10CM:M32 | Systemic lupus erythematosus (SLE) |
|  | **Settings for the performed analyses** | | | |
|  | | Risk analysis | | excluding patients with outcome prior to the time window |
| Sarcoidosis | | | | |
|  | **Outcome definition** | | | |
|  | | Diagnosis | ICD10CM:D86 | Sarcoidosis |
|  | **Settings for the performed analyses** | | | |
|  | | Risk analysis | | excluding patients with outcome prior to the time window |
| Systemic Sclerosis | | | | |
|  | **Outcome definition** | | | |
|  | | Diagnosis | ICD10CM:M34 | Systemic sclerosis [scleroderma] |
|  | **Settings for the performed analyses** | | | |
|  | | Risk analysis | | excluding patients with outcome prior to the time window |
| Psoriasis | | | | |
|  | **Outcome definition** | | | |
|  | | Diagnosis | ICD10CM:L40 | Psoriasis |
|  | **Settings for the performed analyses** | | | |
|  | | Risk analysis | | excluding patients with outcome prior to the time window |
| Sjögren's Syndrome | | | | |
|  | **Outcome definition** | | | |
|  | | Diagnosis | ICD10CM:M35.0 | Sjögren syndrome |
|  | **Settings for the performed analyses** | | | |
|  | | Risk analysis | | excluding patients with outcome prior to the time window |
| Diabetes Type 1 | | | | |
|  | **Outcome definition** | | | |
|  | | Diagnosis | ICD10CM:E10 | Type 1 diabetes mellitus |
|  | **Settings for the performed analyses** | | | |
|  | | Risk analysis | | excluding patients with outcome prior to the time window |
| Autoimmune Thyroiditis | | | | |
|  | **Outcome definition** | | | |
|  | | Diagnosis | ICD10CM:E06.3 | Autoimmune thyroiditis |
|  | **Settings for the performed analyses** | | | |
|  | | Risk analysis | | excluding patients with outcome prior to the time window |

| Crohn's Disease | | | | |
| --- | --- | --- | --- | --- |
|  | **Outcome definition** | | | |
|  | | Diagnosis | ICD10CM:K50 | Crohn's disease [regional enteritis] |
|  | **Settings for the performed analyses** | | | |
|  | | Risk analysis | | excluding patients with outcome prior to the time window |
| Ulcerative Colitis | | | | |
|  | **Outcome definition** | | | |
|  | | Diagnosis | ICD10CM:K51 | Ulcerative colitis |
|  | **Settings for the performed analyses** | | | |
|  | | Risk analysis | | excluding patients with outcome prior to the time window |
| Hypersensitivity Angiitis | | | | |
|  | **Outcome definition** | | | |
|  | | Diagnosis | ICD10CM:M31.0 | Hypersensitivity angiitis |
|  | **Settings for the performed analyses** | | | |
|  | | Risk analysis | | excluding patients with outcome prior to the time window |
| Idiopathic Inflammatory Myopathies | | | | |
|  | **Outcome definition** | | | |
|  | | Diagnosis | ICD10CM:M33 | Dermatopolymyositis |
|  | **Settings for the performed analyses** | | | |
|  | | Risk analysis | | excluding patients with outcome prior to the time window |
| Mixed Connective Tissue Disease | | | | |
|  | **Outcome definition** | | | |
|  | | Diagnosis | ICD10CM:M35.9 | Systemic involvement of connective tissue, unspecified |
|  | **Settings for the performed analyses** | | | |
|  | | Risk analysis | | excluding patients with outcome prior to the time window |
| Celiac Disease | | | | |
|  | **Outcome definition** | | | |
|  | | Diagnosis | ICD10CM:K90.0 | Celiac disease |
|  | **Settings for the performed analyses** | | | |
|  | | Risk analysis | | excluding patients with outcome prior to the time window |
| Autoimmune Hepatitis | | | | |
|  | **Outcome definition** | | | |
|  | | Diagnosis | ICD10CM:K75.4 | Autoimmune hepatitis |
|  | **Settings for the performed analyses** | | | |
|  | | Risk analysis | | excluding patients with outcome prior to the time window |
| Graves' Disease | | | | |
|  | **Outcome definition** | | | |
|  | | Diagnosis | ICD10CM:E05.0 | Thyrotoxicosis with diffuse goiter |
|  | **Settings for the performed analyses** | | | |
|  | | Risk analysis | | excluding patients with outcome prior to the time window |

### **Antinuclear Antibody (ANA) – LOINC code**

|  |  | | | | |
| --- | --- | --- | --- | --- | --- |
|  |  |  | laboratory | LNC:42254-3 | Nuclear Ab [Presence] in Serum by Immunofluorescence (labResult: Positive) |
