# Supplementary material for: New-onset autoimmune disease after COVID-19
Source: Front Immunol. 2024 Feb 8;15:1337406. doi: 10.3389/fimmu.2024.1337406 (PMC10883027; doi:10.3389/fimmu.2024.1337406)
Supplement: Supplementary file 5 [file Table_2.docx]

**Supplemental Table 2. Demographics Overall and by COVID-19 Exposure Group (Secondary Analysis)**

|  | **Overall**  **N=4,407,892** | **COVID-19**  **n=2,203,946** | **No COVID-19**  **n=2,203,946** | **p-value** |
| --- | --- | --- | --- | --- |
| Age at Index | 49.5 (17.9) | 49.5 (17.9) | 49.5 (17.9) | >0.99 |
| Sex |  | | | |
| Female | 2,590,918 (58.8%) | 1,295,459 (58.8%) | 1,295,459 (58.8%) | >0.99 |
| Male | 1,815,994 (41.2%) | 907,997 (41.2%) | 907,997 (41.2%) | >0.99 |
| Unknown | 980 (<1%) | 490 (<1%) | 490 (<1%) | >0.99 |
| Race |  | | | |
| White | 2,509,535 (56.9%) | 1,321,597 (60%) | 1,187,938 (53.9%) | <0.001 |
| Black/African American | 581,945 (13.2%) | 315,204 (14.3%) | 266,741 (12.1%) | <0.001 |
| Asian | 104,261 (2.4%) | 46,313 (2.1%) | 57,948 (2.6%) | <0.001 |
| American Indian, Alaskan Native | 15,155 (0.3%) | 7,977 (0.4%) | 7,178 (0.3%) | <0.001 |
| Pacific Islander | 5,361 (0.1%) | 2,919 (0.1%) | 2,442 (0.1%) | <0.001 |
| Unknown | 1,191,635 (27%) | 509,936 (23.1%) | 681,699 (30.9%) | <0.001 |
| Ethnicity |  | | | |
| Not Hispanic/Latino | 2,468,032 (56%) | 1,327,475 (60.2%) | 1,140,557 (51.8%) | <0.001 |
| Hispanic/Latino | 331,815 (7.5%) | 192,282 (8.7%) | 139,533 (6.3%) | <0.001 |
| Unknown | 1,608,045 (36%) | 684,189 (31%) | 923,856 (41.9%) | <0.001 |

Groups are matched by propensity score. Propensity score includes age, male and female sex. Values shown are mean ± standard deviation for continuous variables and frequency (column percent) for categorical variables. People with any prevalent autoimmune diseases were not excluded prior to propensity score matching.
