# Supplementary material for: New-onset autoimmune disease after COVID-19
Source: Front Immunol. 2024 Feb 8;15:1337406. doi: 10.3389/fimmu.2024.1337406 (PMC10883027; doi:10.3389/fimmu.2024.1337406)
Supplement: Supplementary file 6 [file Table_3.docx]

**Supplemental Table 3. Incident Autoimmune Diseases Overall and by COVID-19 Exposure Group (Secondary Analysis)**

|  | Overall  N=4,407,892 | COVID-19  n=2,203,946 | No COVID-19  n=2,203,946 | Adjusted Risk Ratio  (95% confidence interval) |
| --- | --- | --- | --- | --- |
| Any Autoimmune Disease | 30,267 (0.687%) | 16,164 (0.733%) | 14,103 (0.640%) | 1.22 (1.19-1.25) |
| Autoimmune Diseases More Likely After COVID-19 | | | | |
| Cutaneous Vasculitis | 1,080 (0.025%) | 722 (0.033%) | 358 (0.016%) | 2.02 (1.78-2.30) |
| Polyarteritis Nodosa | 166 (0.004%) | 110 (0.005%) | 56 (0.003%) | 1.97 (1.42-2.71) |
| Mixed Connective Tissue Disease | 2,394 (0.054%) | 1,555 (0.071%) | 839 (0.038%) | 1.86 (1.71-2.02) |
| Hypersensitivity Angiitis | 194 (0.004%) | 123 (0.006%) | 71 (0.003%) | 1.73 (1.29-2.32) |
| Diabetes Mellitus Type 1 | 5,572 (0.126%) | 3,354 (0.152%) | 2,218 (0.101%) | 1.54 (1.46-1.62) |
| Ulcerative Colitis | 3,073 (0.070%) | 1,818 (0.082%) | 1,255 (0.057%) | 1.46 (1.35-1.56) |
| ANCA Associated Vasculitis | 255 (0.006%) | 150 (0.007%) | 105 (0.005%) | 1.43 (1.11-1.83) |
| Psoriasis | 6,855 (0.156%) | 3,916 (0.178%) | 2,939 (0.133%) | 1.35 (1.28-1.41) |
| Idiopathic Inflammatory Myopathies | 363 (0.008%) | 206 (0.009%) | 157 (0.007%) | 1.31 (1.07-1.62) |
| Autoimmune Hepatitis | 482 (0.011%) | 273 (0.012%) | 209 (0.009%) | 1.31 (1.09-1.57) |
| Celiac Disease | 1,633 (0.037%) | 920 (0.042%) | 713 (0.032%) | 1.30 (1.17-1.43) |
| Axial or Peripheral Spondylitis | 817 (0.019%) | 453 (0.021%) | 364 (0.017%) | 1.25 (1.09-1.43) |
| Autoimmune Thyroiditis | 4,209 (0.095%) | 2,316 (0.105%) | 1,893 (0.086%) | 1.23 (1.16-1.31) |
| Systemic Sclerosis | 529 (0.012%) | 290 (0.013%) | 239 (0.011%) | 1.21 (1.02-1.44) |
| Crohn’s Disease | 2,156 (0.049%) | 1,177 (0.053%) | 979 (0.044%) | 1.21 (1.11-1.31) |
| Sjögren Syndrome | 2,630 (0.060%) | 1,430 (0.065%) | 1,200 (0.054%) | 1.20 (1.11-1.29) |
| Rheumatoid Arthritis | 6,856 (0.156%) | 3,666 (0.166%) | 3,190 (0.145%) | 1.17 (1.11-1.22) |
| Polymyalgia Rheumatica | 1,106 (0.025%) | 587 (0.027%) | 519 (0.024%) | 1.13 (1.01-1.28) |
| Autoimmune Diseases with No Associated Increased or Decreased Risk After COVID-19 | | | | |
| Adult Onset Still’s Disease | 54 (0.001%) | 34 (0.001%) | 20 (0.001%) | 1.70 (0.98-2.95) |
| CNS Arteritis | 52 (0.001%) | 32 (0.001%) | 20 (0.001%) | 1.60 (0.92-2.80) |
| Sarcoidosis | 1,326 (0.030%) | 692 (0.031%) | 634 (0.029%) | 1.09 (0.98-1.22) |
| Reactive Arthritis | 58 (0.001%) | 30 (0.001%) | 28 (0.001%) | 1.07 (0.64-1.79) |
| Systemic Lupus Erythematosus | 2,175 (0.049%) | 1,115 (0.051%) | 1,060 (0.048%) | 1.06 (0.97-1.15) |
| Graves' Disease | 1,738 (0.039%) | 863 (0.039%) | 875 (0.040%) | 0.99 (0.90-1.09) |

Groups are matched by propensity score. Propensity score includes age, male and female sex. In this secondary analysis, people with a specific autoimmune disease prior to or within one month after the index date were excluded from the analysis for that outcome only. As a result, n in each group varied slightly for each outcome assessed.
