# Supplementary material for: New-onset autoimmune disease after COVID-19
Source: Front Immunol. 2024 Feb 8;15:1337406. doi: 10.3389/fimmu.2024.1337406 (PMC10883027; doi:10.3389/fimmu.2024.1337406)
Supplement: Supplementary file 7 [file Table_4.docx]

**Supplemental Table 4. Risk of Autoimmune Disease After COVID-19 by COVID-19 Vaccination Status**

|  | Received  Vaccination  n=159,304 | No Documentation  of Vaccination  n=159,304 | Adjusted Risk Ratio  (95% CI) |
| --- | --- | --- | --- |
| Any Autoimmune Disease | 1,633 (1.025%) | 1,383 (0.868%) | 1.18 (1.10-1.27) |
| Celiac Disease | 72 (0.045%) | 40 (0.025%) | 1.80 (1.22-2.65) |
| Autoimmune Thyroiditis | 221 (0.139%) | 130 (0.082%) | 1.70 (1.37-2.11) |
| Sjögren’s Syndrome | 123 (0.077%) | 80 (0.050%) | 1.54 (1.16-2.04) |
| Psoriasis | 366 (0.230%) | 258 (0.162%) | 1.42 (1.21-1.66) |
| Ulcerative Colitis | 150 (0.094%) | 107 (0.067%) | 1.40 (1.09-1.80) |
| Axial or Peripheral Spondylitis | 37 (0.023%) | 23 (0.014%) | 1.61 (0.96-2.71) |
| Systemic Sclerosis | 17 (0.011%) | 13 (0.008%) | 1.31 (0.64-2.69) |
| Systemic Lupus Erythematosus | 66 (0.041%) | 53 (0.033%) | 1.25 (0.87-1.79) |
| Graves' Disease | 65 (0.041%) | 53 (0.033%) | 1.23 (0.85-1.76) |
| ANCA Associated Vasculitis | 12 (0.008%) | ≤10 (0.006%) | 1.20 (0.52-2.78) |
| Idiopathic Inflammatory Myopathies | 13 (0.008%) | 11 (0.007%) | 1.18 (0.53-2.64) |
| Mixed Connective Tissue Disease | 85 (0.053%) | 76 (0.048%) | 1.12 (0.82-1.52) |
| Hypersensitivity Angiitis | 11 (0.007%) | ≤10 (0.006%) | 1.10 (0.47-2.59) |
| Rheumatoid Arthritis | 249 (0.156%) | 244 (0.153%) | 1.02 (0.86-1.22) |
| Crohn’s Disease | 73 (0.046%) | 73 (0.046%) | 1.00 (0.72-1.38) |
| Polyarteritis Nodosa | ≤10 (0.006%) | ≤10 (0.006%) | 1.00 (0.42-2.40) |
| CNS Arteritis | ≤10 (0.006%) | ≤10 (0.006%) | 1.00 (0.42-2.40) |
| Adult Onset Still’s Disease | ≤10 (0.006%) | ≤10 (0.006%) | 1.00 (0.42-2.40) |
| Diabetes Mellitus Type 1 | 246 (0.154%) | 254 (0.159%) | 0.97 (0.81-1.15) |
| Polymyalgia Rheumatica | 42 (0.026%) | 47 (0.030%) | 0.89 (0.59-1.36) |
| Autoimmune Hepatitis | 16 (0.010%) | 19 (0.012%) | 0.84 (0.43-1.64) |
| Sarcoidosis | 53 (0.033%) | 64 (0.040%) | 0.83 (0.58-1.19) |
| Cutaneous Vasculitis | 34 (0.021%) | 45 (0.028%) | 0.76 (0.48-1.18) |
| Reactive Arthritis | 0 (0%) | ≤10 (0.006%) | - |

Groups are matched by propensity score. Propensity score includes age, male, and female sex. People with any prevalent autoimmune diseases prior to or within one month after the index date were excluded from this analysis prior to propensity score matching. All people included in this analysis had COVID-19.
